# Degenerative Ulcerative Colitis After One Year of Evolution in a 20-Year-Old Patient

**DOI:** 10.7759/cureus.18582

**Published:** 2021-10-07

**Authors:** Mouad Ouryemchi, Rachid Jabi, Haitam Soussan, Younesse Najioui, Mohammed Bouziane

**Affiliations:** 1 General Surgery, Mohammed VI University Hospital, Oujda, MAR; 2 Laboratory of Anatomy, Microsurgery and Surgery Experimental and Medical Simulation (LAMCESM), Faculty of Medicine and Pharmacy, Mohammed Ist University, Oujda, MAR; 3 Pathology, Mohammed VI University Hospital, Oujda, MAR; 4 Pathology and Laboratory Medicine, Faculty of Medicine and Pharmacy, Mohammed Ist University, Oujda, MAR

**Keywords:** endoscopy, coloprectectomy, degeneration, colorectal cancer, ulcerative colitis

## Abstract

Ulcerative colitis (UC) is an inflammatory bowel disease. Patients with this condition are considered to belong to a high-risk group for developing colorectal cancer (CRC). These are patients who are often not subjected to regular endoscopic monitoring and in whom the diagnosis of CRC degeneration is often a pathological discovery. The purpose of this work is to report the characteristics of a case of degenerate UC.

This is a case of a 20-year-old patient, followed for UC, who was diagnosed with CRC during a flare-up of his disease, revealed by endoscopic exploration. This patient underwent a coloproctectomy with ileoanal J-pouch reconstruction by laparotomy. The operative specimen came back in favor of a moderately differentiated Lieberkunian adenocarcinoma after an anatomopathological study. The risk of developing CRC in patients followed for UC is rare at a young age, but it becomes higher after 10 years of evolution. This risk is incriminated by several factors: duration of evolution, the extent and severity of inflammatory lesions and the notion of CRC in the family. The discovery is often made by endoscopic exploration during disease surveillance.

## Introduction

Ulcerative colitis (UC) is an inflammatory bowel disease; reaching the rectum and the colon, it most often evolves by relapsing-remitting [1]. UC patients are at increased risk for colorectal cancer (CRC), recent findings suggest that this risk is time-dependent and may increase by 2% after 10 years [2]. Therefore, these patients are enrolled in endoscopic surveillance programs that aim to detect CRC at an early stage [3]. We report the case of a 20-year-old patient, labeled with UC since the age of 17, complicated by CRC after one year of progression, benefiting from surgical resection.

## Case presentation

This is the case of a young man aged 20 years, diagnosed with UC at the age of 17 years, whose symptomatology dates back to three years before his first consultation, where he presented with mucohemorrhagic diarrhea with a rectal syndrome, associated with peripheral arthralgias. The patient underwent a sigmoidoscopy revealing a diffuse erythematous mucosa with irregular ulcerations and pseudo-polyps, the anatomopathological study objectified a UC flare-up, then he was put on 5-aminosalicylic acid (Pentasa®) and corticotherapy.

After one year of evolution marked by a poor therapeutic compliance, the patient reconsulted for a new episode, with Mucohemorrhagic diarrhea and periumbilical pain, in the context of the deterioration of the general state. An abdominopelvic computed tomography scan (CT scan) showed inflammatory parietal thickening involving the entire colonic framework, associated with areas of endoluminal budding tumor thickening 14mm thick in the left and transverse colon. The extension workup came back negative with a biological workup without abnormalities. A colonoscopy with staged biopsies was performed, showing a fragile congestive mucosa bleeding easily on contact, with some superficial ulcerations and multiple polypodal formations, varying in size from 2cm to 7cm. Histology was in favor of UC with tumor proliferation at the level of a polyp of the transverse colon whose type is a moderately differentiated mucinous adenocarcinoma.

**Figure 1:**
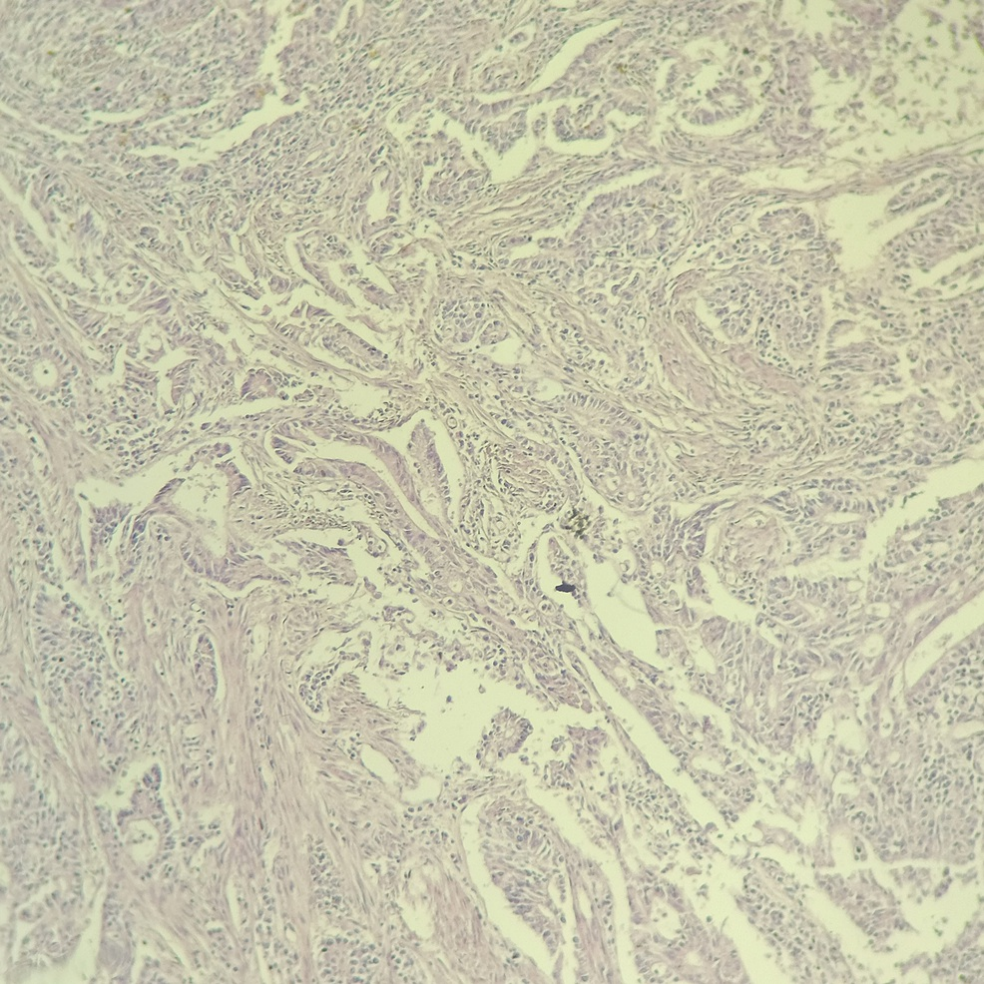
Carcinomatous proliferation, scattered in nests and spans. The tumor cells are atypical, with an irregular and hyperchromatic nucleus; the cytoplasm is eosinophilic.

**Figure 2:**
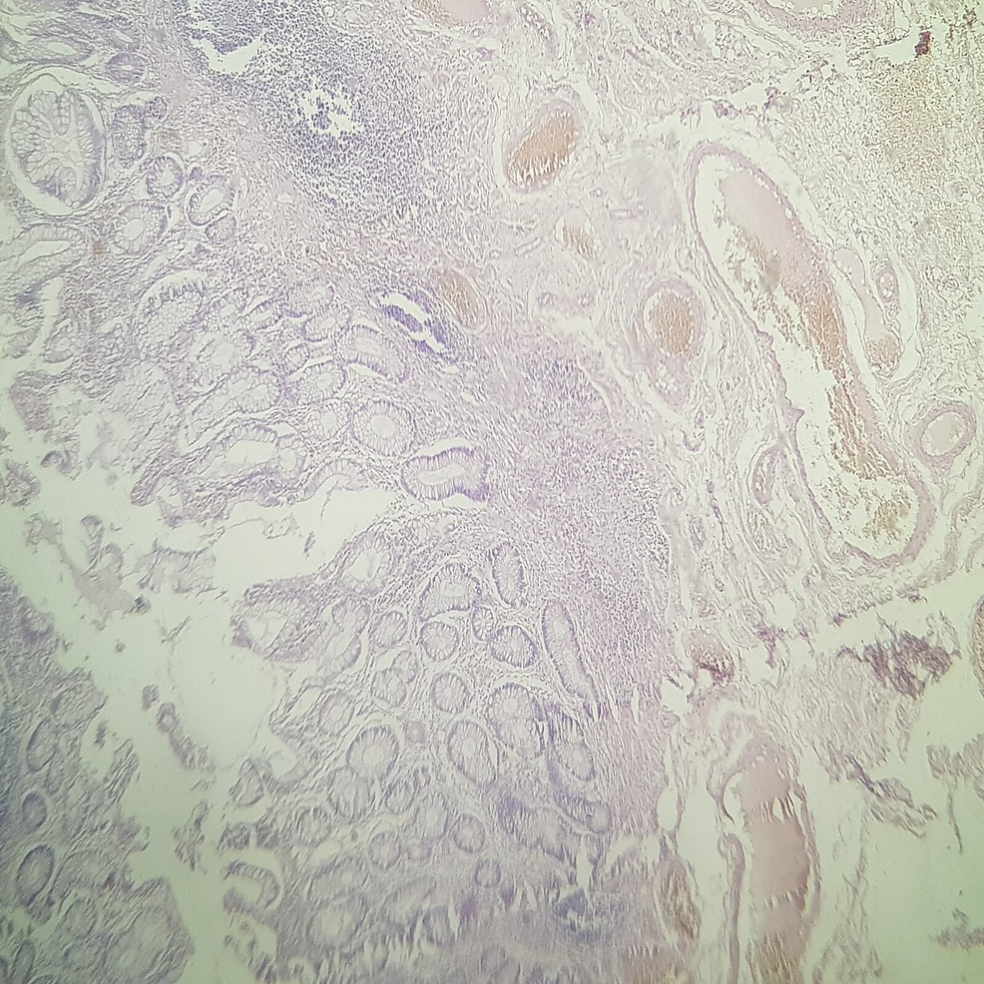
Histological image of ulcerative colitis showing architectural anomalies such as distortion, shortening and bifurcation.

This patient was operated on. At the surgical exploration, we discovered a suspicious process at the level of the transverse colon with lymphadenopathy at the origin of the inferior and superior mesenteric arteries. There was no peritoneal carcinosis or hepatic metastases. A coloproctectomy with ileoanal J-pouch reconstruction was then performed. The anatomopathological study showed a moderately differentiated Lieberkunian adenocarcinoma on the pathological colon, infiltrating the wall up to the subserosa. The surgical resection margins were healthy, without peri-nervous engulfment or vascular emboli. This process was classified as pT3N0M0 (Figures 1, 2).

At D26 postoperatively, the patient was readmitted for generalized abdominal pain with diffuse abdominal sensibility on clinical examination. Laboratory tests showed a hyperleukocytosis of 18,490/µL with a predominance of neutrophils, a C-reactive protein (CRP) of 167mg/L, and ionogram and renal function were normal. The patient underwent an abdominopelvic CT scan, which showed a stercoral peritonitis following a loosening of the ileo-anal anastomosis with individualization of a superinfected collection opposite. The patient was rushed to the operating room. At exploration, a fecal peritoneal effusion of great abundance at the level of the recto-uterine pouch was individualized with pelvic shielding. Thus, an ileostomy was performed, keeping the J-pouch. After three months, this patient benefited from a restoration of continuity.

During a follow-up period spread over four years, a good clinical and biological evolution was noted, regular radiological and endoscopic control was maintained without signs of recurrence or flare-up of his disease.

## Discussion

UC is a chronic inflammatory bowel disease related to an excessive immune response, it usually evolves by flare-ups interspersed with phases of complete remission [1]. The incidence of the disease is highly variable but generally, a peak is found between 15 and 25 years of age and a second peak between 55 and 65 years of age which is inconstant [4]. There is a slight male predominance [5]. The pathophysiology is until now poorly known, moreover, several factors are incriminated, a family genetic predisposition by the presence of family aggregates of UC, active smoking as one of the environmental factors, as well as an immunological factor related to an imbalance between the intestinal immune system and the gut microbiota [4].

The diagnosis of UC is based on a combination of clinical, biological, endoscopic and histological evidence. Clinically, patients usually report an insidious onset of symptoms; chronic diarrhea is often associated with rectorrhagia or traces of blood in the stool. Occasionally, there is a mucusy stool with tenesmus and abdominal pain in the left iliac fossa relieved by defecation [6]. Extra-intestinal manifestations include arthralgia, episcleritis and erythema nodosum, they precede gastrointestinal symptoms in 10% of patients, and are observed in 10%-20% of cases. Biologically, in the subacute phase, a significant disease may exist with normal inflammatory markers. In acute flare-ups, increased white blood cell, platelet or CRP values may be observed. Anemia is seen in 20% of patients. A stool test is essential to rule out an infectious etiology [7]. Sigmoidoscopy, or even total colonoscopy, remains the best endoscopic examination to establish the diagnosis of UC, as well as an evaluation of the extent of lesions in the rectum, sigmoid colon, left colon and right colon. The endoscopic procedure is performed under general anesthesia and on a well-prepared colon [8]. The endoscopic exploration is reinforced by the realization of biopsies along the colon in a comparative way between healthy and diseased areas. The results of the anatomopathological study make it possible to differentiate UC from other colitis.

Most of the time, the lesions are aggravated by successive relapses, increasing the risk of complications. Severe acute colitis is the most frequent complication, affecting 10% to 15% of patients, as well as colonic perforation and severe hemorrhage. CRC complicating UC is rare. It represents 1 to 2% of all cases of CRC. However, the risk of developing CRC during the course of UC is increased compared to the general population. This risk is incriminated by several factors, mainly the duration of evolution (2% at 10 years of evolution, 8% at 20 years and 18% after 30 years) [9], the extent and severity of the intestinal inflammation, primary sclerosing cholangitis, a family history of first-degree CRC, and the appearance of pseudo-polyps during endoscopic exploration. A recent study - based on Swedish and Danish national registers - showed that the early age of diagnosis of UC, especially before the age of 20, is a major risk factor for developing CRC [10]; this is the case with our patient where UC was diagnosed at the age of 17 and has evaluated rapidly after one year.

Often, the diagnosis of CRC in UC is based on surveillance colonoscopy after eight years of progression in affected patients [11]. A biopsy of precancerous lesions or ulcerative burgundy processes for the histological study is necessary to stage the degeneration. It goes through successive stages, inflammatory, low-grade dysplasia, high-grade dysplasia and cancer [12]. The treatment of choice for CRC in UC is surgical, a total coloprotectomy with ileoanal anastomosis is the reference technique for this degeneration whatever its stage.

## Conclusions

Screening and early diagnosis of colon cancer in young UC patients can be improved by educating patients about this risk and by regular and rigorous endoscopic surveillance with systematic biopsies to look for dysplastic lesions. Currently, advances in molecular biology techniques allow defining a group of patients at very high risk of degeneration who are candidates for strict surveillance.
